# A Novel Miniature and Selective CMOS Gas Sensor for Gas Mixture Analysis—Part 4: The Effect of Humidity

**DOI:** 10.3390/mi15020264

**Published:** 2024-02-11

**Authors:** Moshe Avraham, Adir Krayden, Hanin Ashkar, Dan Aronin, Sara Stolyarova, Tanya Blank, Dima Shlenkevitch, Yael Nemirovsky

**Affiliations:** 1 Electrical and Computer Engineering Department, Technion—Israel Institute of Technology, Haifa 3200003, Israel; smoa@campus.technion.ac.il (M.A.); adir-krayden@campus.technion.ac.il (A.K.); haninashkar@campus.technion.ac.il (H.A.); aronindan@campus.technion.ac.il (D.A.); ssstolya@g.technion.ac.il (S.S.); tblank@technion.ac.il (T.B.); 2 TODOS Technologies, Airport City 7019900, Israel; dima@todos-technologies.com

**Keywords:** gas sensor, pellistor, CFD, TMOS, GMOS, humidity

## Abstract

This is the fourth part of a study presenting a miniature, combustion-type gas sensor (dubbed GMOS) based on a novel thermal sensor (dubbed TMOS). The TMOS is a micromachined CMOS-SOI transistor, which acts as the sensing element and is integrated with a catalytic reaction plate, where ignition of the gas takes place. The GMOS measures the temperature change due to a combustion exothermic reaction. The controlling parameters of the sensor are the ignition temperature applied to the catalytic layer and the increased temperature of the hotplate due to the released power of the combustion reaction. The solid-state device applies electrical parameters, which are related to the thermal parameters. The heating is applied by Joule heating with a resistor underneath the catalytic layer while the signal is monitored by the change in voltage of the TMOS sensor. Voltage, like temperature, is an intensive parameter, and one always measures changes in such parameters relative to a reference point. The reference point for both parameters (temperature and voltage) is the blind sensor, without any catalytic layer and hence where no reaction takes place. The present paper focuses on the study of the effect of humidity upon performance. In real life, the sensors are exposed to environmental parameters, where humidity plays a significant role. Humidity is high in storage rooms of fruits and vegetables, in refrigerators, in silos, in fields as well as in homes and cars. This study is significant and innovative since it extends our understanding of the performance of the GMOS, as well as pellistor sensors in general, in the presence of humidity. The three main challenges in simulating the performance are (i) how to define the operating temperature based on the input parameters of the heater voltage in the presence of humidity; (ii) how to measure the dynamics of the temperature increase during cyclic operation at a given duty cycle; and (iii) how to model the correlation between the operating temperature and the sensing response in the presence of humidity. Due to the complexity of the 3D analysis of packaged GMOS, and the many aspects of humidity simultanoesuly affecting performane, advanced simulation software is applied, incorporating computational fluid dynamics (CFD). The simulation and experimental data of this study show that the GMOS sensor can operate in the presence of high humidity.

## 1. Introduction

### 1.1. SMO Sensors and GMOS Sensors

In recent years, the need for mobile, low-cost and low-power gas sensors has increased dramatically. Such gas sensors are needed for safety in homes and cars, monitoring air quality, the well-being of people as well as industrial process control and sustainable smart agriculture [1,2,3,4,5,6,7,8]. During the past few decades, SMO (Semiconductor Metal Oxide, also referred to as MOX) gas sensors have become a leading technology in domestic, commercial and industrial gas-sensing systems because of their following features: low cost, adequate sensitivity and easy to measure response (a change in resistivity). However, these sensors have problems in reproducibility, stability and selectivity.

### 1.2. GMOS Sensor

A completely different class of gas sensors, known as pellistors, measures the temperature change due to a combustion exothermic reaction. Traditional pellistor sensors use a thermal sensor to measure temperature change. The sensor is usually a resistor, but thermopiles and pyroelectric ceramics have also been reported.

**Figure 1:**
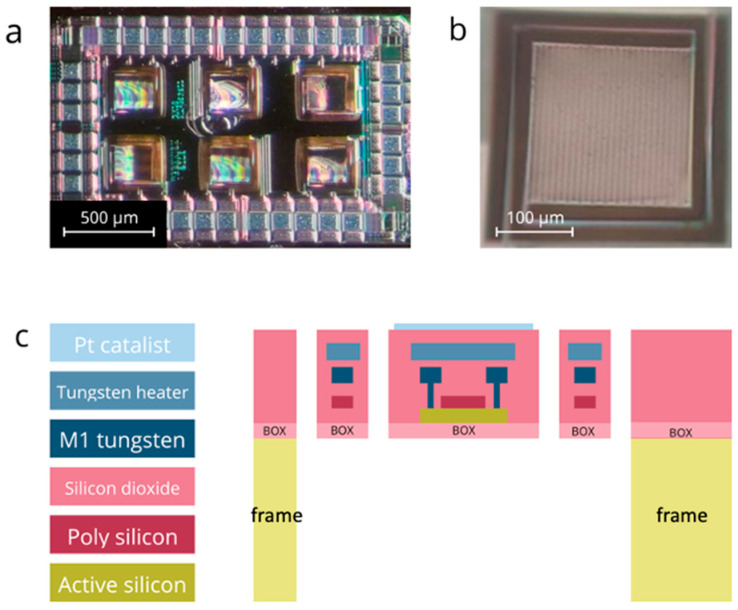
(**a**) The microscopic image of the GMOS sensing die with six sensing pixels. The die size is 2000 μm × 1380 μm; (**b**) The microscopic image of a single sensing pixel. The pixel size is 213 μm × 213 μm. (**c**) The cross-section scheme of the GMOS sensing pixel deposited above the buried oxide (BOX). The figure is from [15].

In the last decade, a thermal sensor (dubbed TMOS) based on CMOS-SOI technology has been studied and reached maturity; it is now mass-produced in a commercial fab [9,10]. The sensing element is a suspended MOSFET transistor (Figure 1a) that operates at subthreshold, and therefore requires low power consumption.

These advantages of TMOS have been applied to fabricate a new combustion-type, pellistor-like gas sensor (dubbed GMOS). To fabricate a GMOS, an integrated heating resistor is added to a TMOS thermal sensor (Figure 1b) and the catalytic layer is applied on the top surface of the pixel. The feasibility of GMOS sensing and its advantages have been reported in several publications [11,12]. In contrast to SMO or MOX sensors, where the sensing element interacts with the gas, the GMOS sensing element—the micromachined CMOS transistor—does not interact directly with the gas, and therefore has potential for long-term stability.

The present paper complements the series of papers published in micromachines analyzing the innovative GMOS sensor [13,14,15]. In the first two previous papers, the sensing [13] and electrical [14] properties of this sensor were described. The third paper focused on the investigation of the sensing mechanism, modeling the underlying thermodynamic and chemical processes, by applying advanced tools for 3D simulation of fluid dynamics [15].

This study focuses on the effect of humidity on the performance of GMOS. The sensors are calibrated in the lab. However, in real life, the sensors are exposed to environmental parameters, where humidity plays a significant role. Humidity is high in the storage rooms of fruits and vegetables, in refrigerators, in silos, in fields as well as in homes and cars. Humidity affects the thermal conductivity and thermal diffusivity of air as well as other thermophysical properties and the enthalpic heat of combustion of the analyte, and accordingly the simulation and 3D modeling of the GMOS are revisited in this study.

Due to the complexity of the 3D analysis of packaged GMOS, and the many aspects of humidity simultaneously affecting performance, advanced simulation software is applied, incorporating computational fluid dynamics (CFD). The implementation of CFD modeling provides valuable data about the thermal properties of the investigated device. The simulation and experimental data of this study exhibit that the GMOS sensor can operate in the presence of high humidity.

This paper is organized as follows. The thermophysical properties of humid air are reviewed in Section 2. In Section 3, the static and dynamic operation of the GMOS designed with a bridge-like readout in the presence of humidity are revisited. The simulation results are presented in Section 4, followed by the experimental results in Section 5. Finally, Section 6 concludes the paper.

## 2. The Thermophysical Properties of Humid Air

Humid air has different thermal transport properties compared to dry air and this affects the static as well as dynamic response of gas sensors. The thermophysical properties of humid air at the temperature range between 0 and 100 °C as well as at higher temperatures, as a function of humidity and temperature, are required for ordinary heat transfer engineering calculations.

**Figure 2:**
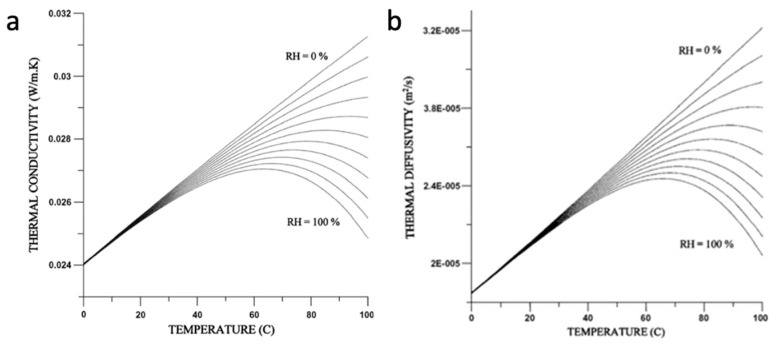
In the presence of moisture, (**a**) the thermal conductivity of air decreases; similarly, (**b**) the thermal diffusivity of air decreases. The decrease starts at approximately 40 °C and the effect becomes more pronounced as temperature and moisture increase. Figures obtained from [16] with permission.

An investigation into the thermophysical and transport properties of humid air at a temperature range between 0 °C and 100 °C based on simulation can be found in [16]. A recent review paper on CMOS thermal sensors supports the data of the earlier paper [17], as shown in Figure 2.

Table 1 is an overview of the main physical parameters required for the modeling and simulation of the GMOS sensors in the presence of humidity.

Figure 1 shows how the thermal conductivity and diffusivity of air change with temperature for different values of relative humidity. It shows that for a given temperature, the specific thermal conductivity decreases significantly above 50° C as humidity increases. A maximum value of thermal conductivity is developed for each fixed relative humidity curve, which moves towards higher temperatures as the relative humidity decreases. This maximum typically moves from the temperature of 60 °C to about 95 °C as the relative humidity (RH) decreases from saturation-level conditions to about RH = 40%.

These results can be understood if we pay attention to the fact that the thermal conductivity of water vapor is lower than that of air. To really feel intuitively why at higher temperatures there is a much stronger effect of humidity, we need to remember that air can hold much larger amounts of water vapor at higher temperatures. If in the gas box at the lab there is a certain amount of water vapor, as temperature increases, the relative humidity decreases.

For this reason, humidity compensation of the measurements becomes especially important as temperature increases. To compensate for this effect, a separate humidity sensor is integrated into the sensing system, hoping that with the right algorithm we can take this effect into account.

Table 1 indicates that the thermal heat capacitance of water vapor is larger than that of dry air. This tells us that the presence of humid air will affect the transient response of the sensor and cause a decrease in the maximum achievable temperature. Accordingly, we need to re-visit the static and dynamic modeling of the GMOS sensor to consider the effect of the parameters of Table 1.

## 3. Revisiting the Static and Dynamic Operation of the GMOS Designed with a Bridge-like Readout in the Presence of Humidity

### GMOS Sensor

The oxidation reaction of the sensed gas is ignited at a given temperature, which is specific for the measured gas and the catalytic layer (denoted below by T*—see references [11,15]). To achieve the ignition temperature, the catalytic layer’s temperature is increased by Joule heating dissipated by the resistor in contact with the reaction plate. The value of the required resistor is determined by the following heat flow equation:

(1) $$C_{th}\frac{d\left(T-T_{0}\right)}{dt}+G_{th}\left(T-T_{0}\right)=P_{Joule-heating}+P_{reaction}$$

where, at steady state,

(2) $$T=T_{0}+\Delta T_{J}+\Delta T_{R}=T_{0}+\frac{P_{Joule-heating}}{G_{th}}+\frac{P_{reaction}}{G_{th}}$$

$T_{0}$ is the frame temperature (see Figure 1c), which is determined by the ambient temperature and

(3) $$P_{Joule-heating}=I^{2}R(T)=\frac{V^{2}}{R(T)}$$

We apply voltage *V* to avoid thermal runaway, since the heating resistor, denoted by *R*(*T*), has positive TCR (Temperature Coefficient of Resistance). See Section 4.

$P_{reaction}$ is the power released by the exothermic oxidation reaction [11].

(3a) $$\Delta T_{reaction}=\frac{P_{reaction}}{G_{th}}$$

**Figure 3:**
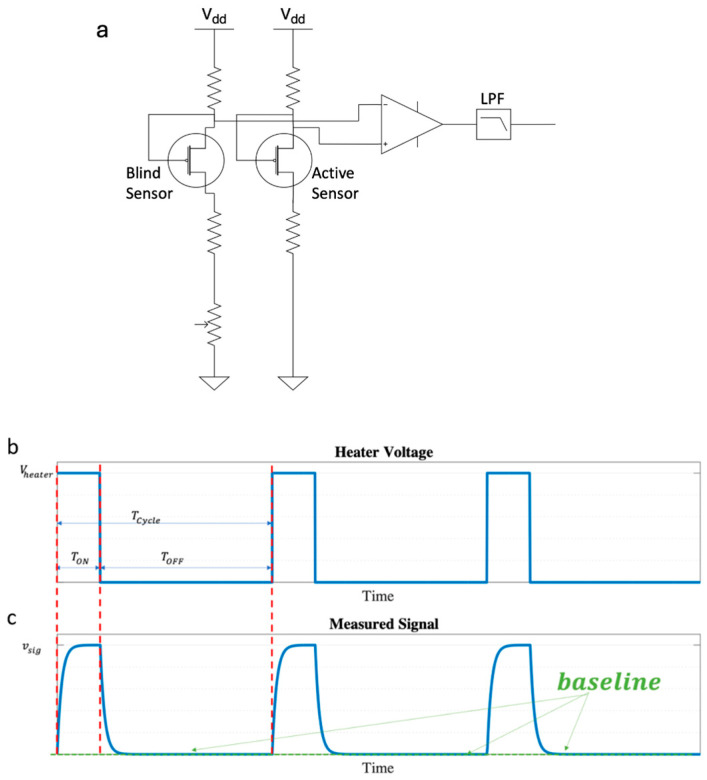
(**a**) Schematics of the differential, bridge-like circuit controlling the operation point at subthreshold; (**b**) the heating cycle imposed by the circuit in the form of periodic pulses, denoted by Tcycle; (**c**) the measured signal as a function of time correlates with the heater voltage pulses. The measured signal is defined relative to the baseline, which is presented. The vertical dashed red lines relate the data of (**b**,**c**) in the same time interval.

At steady state, in the absence of a reaction, the Joule heating increases the TMOS temperature as follows:

(3b) $$\Delta T_{J}=\frac{I\cdot V}{G_{th}}=\frac{I^{2}\cdot R(T)}{G_{th}}=\frac{V^{2}}{R(T)\cdot G_{th}}$$

where R(T) is the resistor heating the reaction plate and *V* is the applied voltage to the heater. Equation (3) enables us to design the required resistor value, which will achieve the specific ignition temperature denoted by T* for each measured gas. $G_{th}$ is a function of both temperature and humidity. At this point, we remind the reader that the Joule heating required to ignite the TMOS to its ignition temperature (above 100 °C) is far larger than the power released by the exothermic reaction, which is typically below 1 °C for low concentrations of analytes. Therefore, a differential electrical readout is mandatory, as described below (see Figure 3).

**Figure 4:**
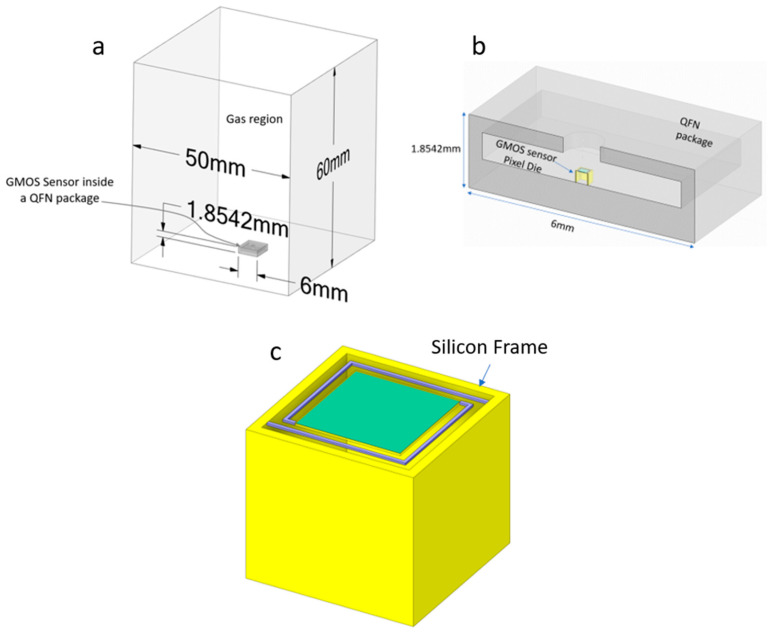
The model for simulation: (**a**) The gas region including the QFN (Quad Flat Non-Lead) package, simulating the gas box at the lab only smaller to reduce simulation time. (**b**) A cross-section of the QFN package where the GMOS sensor is mounted. The QFN dimensions correspond to the commercial chip carrier, which we use. (**c**) The modeled single pixel of the GMOS sensor die, where the frame is shown. The top layer is the micromachined reaction plate and held with two arms to conserve planarity.

The GMOS signal voltage is directly related to the change in the output voltage of the TMOS transistor operating at a subthreshold level and the temperature increase ∆*T* obtained from the power released by the exothermic oxidation of the analyzed gas:

(4) $$v_{sig}=\Delta T{\left(\frac{dV_{DS}}{dT}\right)}_{I_{op};T_{op}}=\frac{P_{reaction}}{G_{th,eff}}{\left(\frac{dV_{DS}}{dT}\right)}_{I_{op};T_{op}}$$

where $\frac{dV_{DS}}{dT}$ is the temperature sensitivity of the TMOS sensor at a given operation point and temperature, $I_{op}$ and $T_{op}$, respectively [11,13]. The above analysis describes the relation between the temperature parameters that control the performance of the thermal sensor and the voltage parameters that are induced and measured. At this point, we remind the reader that temperature and voltage are both intrinsic parameters (following the thermodynamic definition) and must be defined relative to a reference point. For a single pixel, the reference is the frame, which is held at *T*_0_ and is determined by the ambient temperature and humidity. The frame is shown in Figure 1c and Figure 4c.

The differential readout concept is shown in Figure 3.

The front-end analog readout is based on a “bridge-like” differential circuit, which measures the voltage difference between the GMOS sensor with the catalytic layer (dubbed “active”) and an identical micro-machined TMOS without it (dubbed “blind”). The differential approach removes the baseline DC current, allowing higher dynamic range and higher accuracy. It removes the baseline drift—a major issue in the field of gas sensors [18,19].

Furthermore, since the TMOS sensor which measures the temperature increase is operated at a subthreshold level, another major challenge is to control the operation points (V-out 1 and V-out 2). The bridge-like readout addresses this issue as well. A calibration block corrects the residual mismatch between the active and blind pixels and provides the required small DC current (tens of nano-amps) needed to bring the front-end output to a mid-supply level. Calibration is performed at Joule power up and down (Joule power off) and within constant time intervals, as needed. The differential readout enhances sensitivity and selectivity and reduces drift and aging.

We now repeat the modeling by referring to what we apply and measure in the differential mode, namely, using the “blind” sensor as the reference.

(5) $${\left(T-T_{0}\right)}_{active}=∆T_{J}+∆T_{R}=\frac{P_{Joule-heating}}{G_{th}}+\frac{P_{reaction}}{G_{th}}\;{\left(T-T_{0}\right)}_{blind}=∆T_{J}=\frac{P_{Joule-heating}}{G_{th}}\;{\left(T-T_{0}\right)}_{active}-{\left(T-T_{0}\right)}_{blind}={\left[\frac{P_{joule-heating}}{G_{th}}\right]}_{active}-{\left[\frac{P_{joule-heating}}{G_{th}}\right]}_{blind}+{\left[\frac{P_{reaction}}{G_{th}}\right]}_{active}$$

We now assume that the *T*_0_ of both the active and blind sensors is the same (the frame is common to both pixels). We also assume that $G_{th}$ is the same since it is determined by the air and the environment (see simulations in Section 4).

Accordingly,

(6) $$T_{active}-T_{blind}={\left[\frac{P_{reaction}}{G_{th}}\right]}_{active}$$

Assuming that the electrical circuit compensates for the mismatch between the two heating channels, an increase in temperature due to the reaction is obtained.

In the absence of a reaction, we calibrate the baseline, which is the first two terms of the right side of (5). Equation (6) is affected by humidity since $G_{th}$ is affected by humidity. However, the simulations and measurements indicate that $G_{th}$ is determined by the ambient temperature, which is less than 50 °C in our measurements. Hence, the effect of humidity on Equation (6) is negligible.

During this calibration, when we apply the voltage step, the pixel follows the transient heat equation.

(7) $$C_{th}\frac{d\left(T-T_{0}\right)}{dt}+G_{th}\left(T-T_{0}\right)=P_{Joule-heating}$$

where $C_{th}$ is the thermal capacitance of the device. At *t* = 0, the first term of (7) is dominant, and we observe a “jump” in the temperature:

(8) $$\begin{array}{l} C_{th}\frac{d\left(T-T_{0}\right)}{dt}\sim \frac{V^{2}}{R(T)Gth}\\ \frac{d\left(T-T_{0}\right)}{dt}\sim \frac{V^{2}}{Cth\times R(T)Gth} \end{array}$$

The decay time is determined by the thermal parameters of the pixel, namely, $C_{th}$ and $G_{th}$. Since the $C_{th}$ of humid air is larger than that of dry air, the decay towards steady state is slower in the presence of humidity, as confirmed by the measured results (see Section 5).

Once we introduce the gas, and there is an exothermic reaction, the active pixel temperature is slightly elevated, and the measured signal above the baseline is

(9) $$∆T=T_{active}-T_{blind}={\left[\frac{P_{reaction}}{G_{th}}\right]}_{active}\;∆V_{sig}=\left(∆T\right){\left(\frac{dV_{output}}{dT}\right)}_{I_{op};T_{op}}=\frac{P_{reaction}}{G_{th}}\;{\left(\frac{dV_{output}}{dT}\right)}_{I_{op};T_{op}}$$

As discussed above, a calibration is performed for each applied heating voltage to null the temperature difference of 5, while there is no reaction.

## 4. Simulation Results

The simulation tool is ANSYS Fluent 2022a [20]. The parameters of Table 2 and Table 3 are mainly based on the properties reported in the software’s library of materials.

The boundary conditions are imposed on the edges of the structure shown in Figure 4. A gas chamber with dimensions of 50 mm × 50 mm × 60 mm was modeled as shown Figure 4a. The bottom of the chamber was designated as a “wall” boundary with a fixed temperature of 300 K. The sides of the chamber were defined as “pressure inlet” boundaries, while the top was designated as a “pressure outlet” boundary. For simulations involving humid air, the thermal properties of the sides and top were set to a constant temperature of 300 K and the desired mass fraction of water vapor.

The Quad Flat Non-Lead (QFN) package, comprising lead and housing the GMOS sensor, was modeled as illustrated in Figure 4b. The dimensions of the package are 6 mm × 6 mm × 1.85 mm. A constant temperature boundary condition of 300 K was enforced on the bottom surface of the package.

Within the Fluent software environment, the Energy model was activated to facilitate thermal simulation. Furthermore, the Species Transport model was activated to capture the behavior of humid air within the computational domain. To optimize computational resources and execution time, only a single pixel of the GMOS sensor die was simulated within the QFN package. The Joule heating generated by the heating resistor was modeled as a volumetric heat source, with the power dynamically adjusted based on the heater resistance. This dynamic adjustment was necessary due to the temperature-dependent variation in the resistance of the heating element.

**Figure 5:**
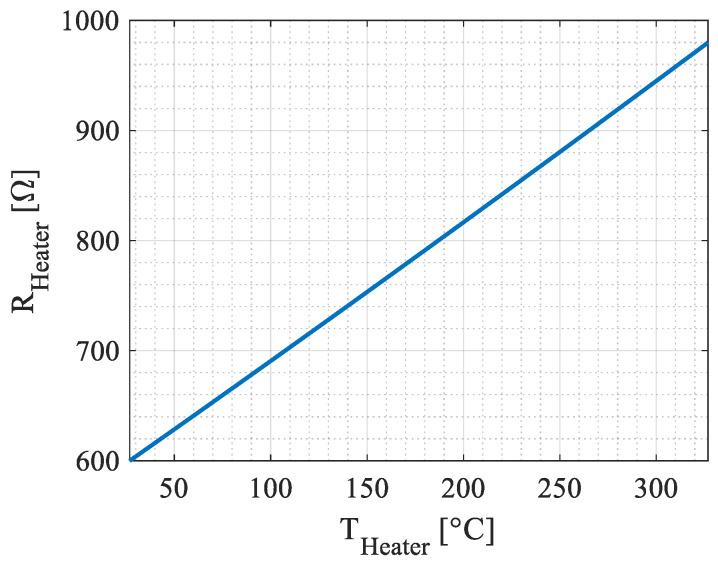
The tungsten heater resistance value as function of the resistor temperature. The TCR (Temperature Coefficient of the Resistor) is given by the fab that manufactured the dies [21].

Figure 5 exhibits the tungsten heating resistor value as function of the temperature:

*R*(*T*) is given by (10)

(10) $$R_{heater}\left(T\right)=R_{0}\left(1+TCR_{1}\cdot \left(T-T_{0}\right)+TCR_{2}\cdot {\left(T-T_{0}\right)}^{2}\right)$$

where *TCR*_1_ = 2.05 × 10^−3^; *TCR*_2_ = 0.2 × 10^−6^.

**Figure 6:**
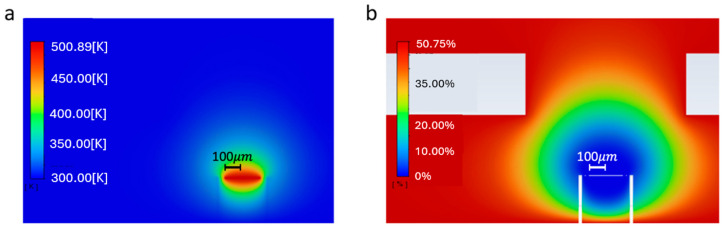
Steady-state simulation results: (**a**) Temperature; blue is around 300 K; the temperature is increased only in the vicinity of the heating resistor on the device; the color bar of (**a**) represents the temperature of the heated membrane (the highest temperature represented by the red color at the edge 500.89 K) and the blue color represents the temperature of the ambient at 300 K. (**b**) Relative humidity; red is around 50% relative humidity; in the vicinity of the packaged device, the relative humidity is very low because of the high temperature imposed on the reaction plate. The color bar of (**b**) represents the relative humidity near the heated membrane (the lowest relative humidity represented by the blue color at the edge 0.07%) and the red color represents the relative humidity in the ambient at 50.75%.

Leveraging the capabilities of Ansys Fluent, this research utilizes computational fluid dynamics (CFD) simulations to model and analyze the temperature, relative humidity within the fluid/gas region, and the heat generated by the gas reactions. Figure 6 illustrates the steady-state results of the simulation for an applied heater voltage of 3 V and a relative humidity of 50% in the surrounding environment.

### 4.1. Steady-State Simulations

**Figure 7:**
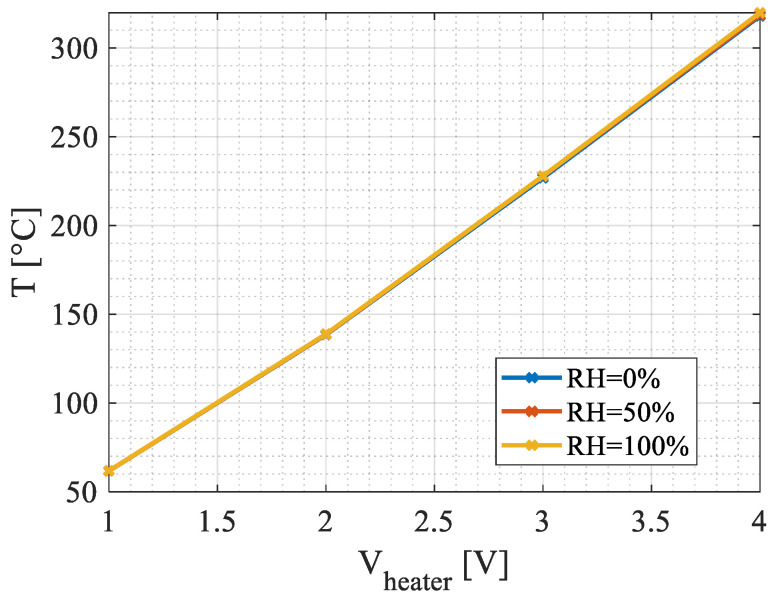
The reaction plate temperature of the active pixel as function of the heater voltage and the environment’s relative humidity. The effect of humidity in the packaged device is small—less than 0.5 K, as shown in higher applied ignition temperatures (above 3 V).

Thermal properties of the reaction membrane were determined through the analysis of simulation results. Figure 7 depicts the steady-state maximum temperature of the plating reaction of the active pixel as a function of the applied heater voltage.

For a given applied heater voltage, at steady state, the temperature hardly increases with humidity. This is explained below. A crucial thermal parameter for the device is its thermal conductance, denoted by $G_{th}$. This parameter quantifies the ease with which heat flows through the device. A lower thermal conductance results in a higher heating temperature for the thermal sensor, leading to improved responsivity.

Under steady-state conditions, the thermal conductance can be expressed as follows:

(11) $$G_{th}\left(T\right)=\frac{P\left(T\right)}{∆T}\left[\frac{W}{K}\right]$$

**Figure 8:**
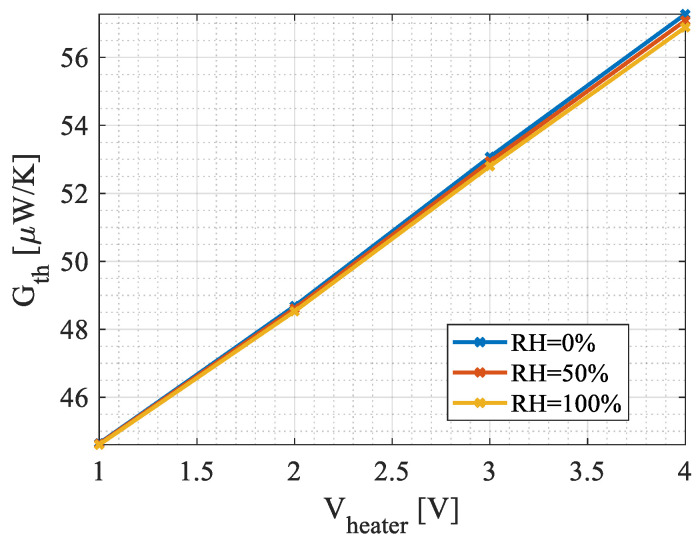
The device’s thermal conductance as function of the heater voltage and the environment’s relative humidity. *Gth* increases with temperature. At the highest heating voltage, *Gth* is slightly reduced as the relative humidity increases.

Leveraging the simulation results, the thermal conductance of the device was determined. Figure 8 presents the values calculated by simulations. As expected, $G_{th}$ increases with temperature. As explained in Section 2, for a given heater voltage and hence temperature, $G_{th}$ is slightly reduced if humidity increases.

### 4.2. Transient Simulations

The transient response of a thermal sensor is well established and is like the exponential charging of a capacitor since temperature is an intrinsic parameter (according to the thermodynamic concept) like voltage.

**Figure 9:**
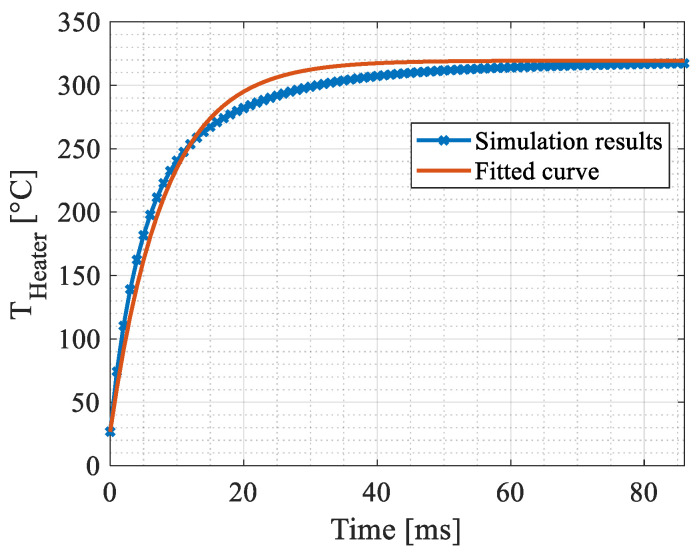
Transient simulation results of the heater temperature. The applied voltage was set to 4 [V] and the environmental relative humidity was set to 50%.

The transient response of the device is approximated by the following equation:

(12) $$T\left(t\right)=\frac{P\left(T\right)}{G_{th}\left(T\right)}\left(1-e^{-\frac{t}{\tau_{th}}}\right)+T_{0}$$

where $T_{0}$ is the initial temperature of the device at *t* = 0, and $\tau_{th}$ is the thermal time constant/response of the device. At the steady state, $T-To=P(T)/G_{th}(T)=292.6\;[K]$; therefore, with the above equation, the thermal time constant is evaluated from the transient simulation results. From Figure 9, the thermal time constant is ~8 [ms]. It should be noted that this simulation only relates to humid air stabilization and does not include a combustion reaction.

The thermal time constant can be estimated by $C_{th}/G_{th}$ and is given by

(13) $$\tau_{th}=\frac{C_{th}}{G_{th}}$$

where $C_{th}$ is the thermal capacitance of the device.

**Figure 10:**
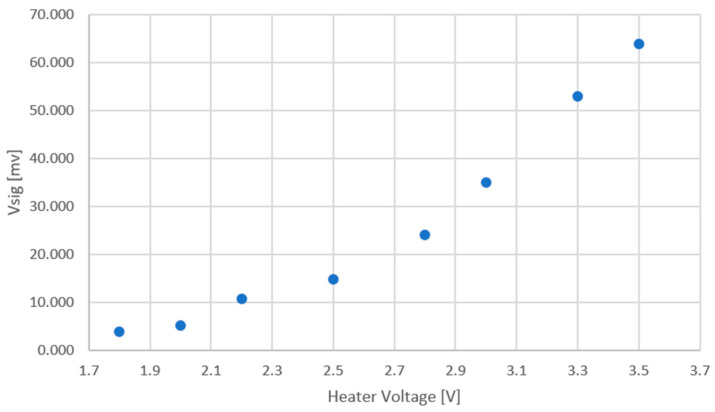
Measured results for 100 PPM of ethanol as an example gas, in the presence of moderate humidity of 50%. The signal voltage is plotted against the heater voltage.

**Figure 11:**
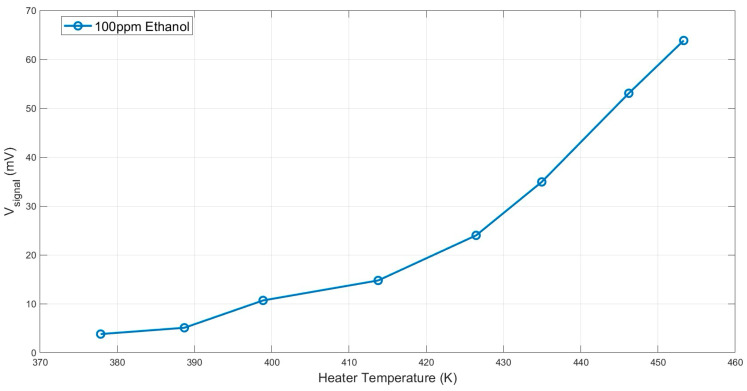
Measured results for 100 PPM of ethanol as an example gas, in the presence of moderate humidity of 50%, as a function of the heater temperature.

**Figure 12:**
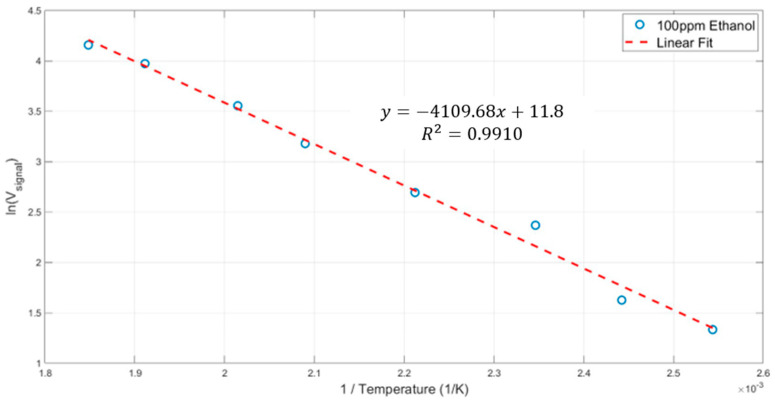
ln(V) signal as a function of 1/T yielding the energy of activation for ethanol.

**Figure 13:**
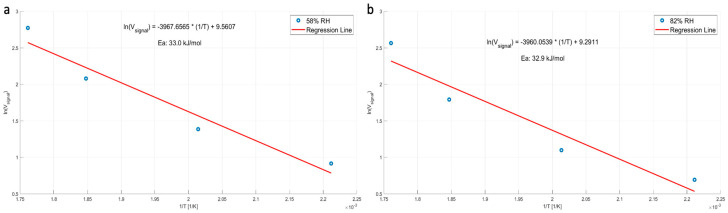
ln(V) signal as a function of 1/T yielding the energy of activation for 30 PPM of ethylene: (**a**) 58% relative humidity; (**b**) 82% relative humidity. The activation energy is practically the same at both humidity values. The measurements were performed at ambient (lab) temperature of 23 °C.

The operating temperature is an important feature of many gas sensors. The precise detection and control of the heater temperature is crucial for an understanding of the sensor, as this is one of the main parameters defining the sensing response (see Figure 3c). The highly sensitive temperature sensor (TMOS) implemented in the GMOS setup as a sensing element can also be used as thermometer for the detection of operating temperature. The simulated heater temperature as a function of Joule heating voltage, for various humidity values, is shown in Figure 7. These results are presented in Figure 10, Figure 11, Figure 12 and Figure 13, shown below.

## 5. Measured Results

The measurements were performed in 6 L hermetic chamber. The humidity was maintained by a Petri dish filled with water and placed inside the chamber next to the GMOS sensing system, including gas, temperature, and humidity sensors. The gases were introduced to the chamber from calibrated gas/air mixtures (provided by GasTech, Zoringen, Germany) upon stabilization of the humidity level. The sensing procedure was repeated after the removal of water and ventilation of the chamber. The measurements were performed at an ambient (lab) temperature of 23 °C. The GMOS pixel temperatures varied from 452 K to 567 K using heater voltages of 2.5, 3.0, 3.5 and 3.8 V.

In the measurement setup described above, the temperature increase of the TMOS is translated into $v_{sig}$, while the heater temperature is determined by the voltage applied to the heating resistor denoted by $V_{heater}$ depending on the humidity (see Figure 7). The value of $v_{sig}$ is directly proportional to the gas concentration $C_{g}$, while the ignition temperature (T*) is specific to the gas for a given catalytic layer, thus enabling both the identification as well as concentration determination of the analyzed gas.

Figure 10 exhibits the measured results for 100 PPM of ethanol as an example gas, in the presence of moderate humidity of 50%.

Based on the simulation, the heater temperature on the x axis is now presented in Figure 11.

Such measurements enable us to determine the energy of activation for ethanol combustion with the ink under study.

We assume that we are in the surface reaction regime and that the measured voltage signal is determined by the Arrhenius formula. The slope yields Ea/R, where R = 8.314 J/mol·K.

The slope of 4109 × R yields Ea = 34 kJoule/mole for the ink under study. The reported Ea value of ethanol upon introduction to the metallic Pt catalyst is ~54 KJ/mol. As expected, the nanoparticle ink outperforms the metallic platinum. The energy of activation is not affected by the humidity or concentration. This is a remarkable characteristic of the GMOS sensor. It should be noted that Pt ink is not the best catalytic layer for ethanol. Ethanol is just an example gas.

Ethylene was measured and analyzed in the same manner, yielding an energy of activation of 33 kJ/mole at low and high humidity, as shown in Figure 13, for a concentration of 30 ppm.

## 6. Summary and Conclusions

The main research problem of this study is focused on the effect of humidity on the innovative catalytic combustion pellistor-like gas sensor, dubbed GMOS. This study is important since in real-life applications, the sensor’s performance is expected to not be affected by humidity. Humid air has different thermal transport properties compared to dry air and this affects the static as well as the dynamic response of all gas sensors.

To gain an intuitive understanding of the many parameters that affect GMOS performance, we first reviewed the thermophysical properties of humid air. Surprisingly, the Web (World Wide Web) reports conflicting results and limited information for the temperature and humidity dependence of gas mixtures (dry air and water vapor) upon density, viscosity, specific heat capacity, thermal conductivity, and thermal diffusivity at total barometric pressure of 1 atmosphere [22]. Ref [22] is not new but it describes the status of the insufficient data that exists even today.

The detection mechanism of commercial chemical gas sensors relies on the chemical reaction between a gas and chemically active material. Semiconductor Metal Oxides (SMOs, also referred to as MOX), which are the most prevalent gas sensors, are affected by humidity, resulting in high drift, since the sensing oxide adsorbs water vapors [6,7,17]. Filters may be used to mitigate this issue, but this approach increases sensor cost and is never dependable for long-term service.

The problem of chemical sensor stability over time is often known as “sensor drift”. It consists of non-deterministic temporal variations in the sensor’s response when it is exposed to the same analytes under identical conditions. Even in commercially available MOX sensors, the drift phenomenon is still not totally understood since several contributing aspects are simultaneously present. However, as discussed above, a major cause of drift is the fact that SMO or MOX sensors adsorb water vapors [18,19].

This study presents the advantages of the GMOS sensor. The printed catalytic layer does not adsorb water and its response is stable in a wide range of humidities. We have studied the effect of humidity both experimentally as well as by simulation, in a vacuum-tight gas box, using ethanol and ethylene as the example gases. It is experimentally shown that the measured activation energy of the printed ink does not depend on humidity, thus proving that it is inert to this effect.

Humidity does affect the correlation between the applied heating voltage and the ignition temperature at high temperatures. This results in a temperature bias between the active and blind sensors. This affects the baseline of the sensor and can be solved by an optimized circuit design.

In summary, the main research problem in this study is to demonstrate the performance of the GMOS sensor in the presence of high humidity. Humidity affects the thermophysical properties of the gas under study in a conflicting and non-intuitive manner. Moreover, the overall performance of the GMOS sensor depends on many parameters, such as the readout circuit, electrical operation point, and the printed ink, as discussed in the previously reported parts. Therefore, we study here the effect of humidity by advanced simulation software, incorporating Computational Fluid Dynamics (CFD). The simulation is validated by analytical modeling and by experiments. The bottom line of this study is that pellistor-like GMOS sensors can perform well even in the presence of humidity. Finally, a caveat: Please note that electronics hardware is not tolerant of high humidity. PCBs manufacturers offer special protective coatings to address this issue.

## Figures and Tables

**Table 1:** Typical thermophysical parameters of dry air and water vapors.

Parameter	Description	Dry Air at 300 K	Water Vapor at 300 K	Note
k $\left[\frac{W}{m\cdot K}\right]$	Thermal conductivity	0.026	0.023	Lower for water vapor
α$\left[\frac{m^{2}}{s}\right]$	Specific thermal diffusivity	$1.9\times 10^{-5}$	$1.2\times 10^{-5}$	Lower for water vapor
$c\;\left[\frac{J}{Kg\cdot K}\right]$	Specific heat capacity	1006.43	1857.72	Higher for water vapor
ρ [Kg/m^3^]	Density	1.225	0.5542	As temp. increases, the density decreases

**Table 2:** The physical properties of the gas.

	k [W/m·K]	C [J/Kg·K]	Viscosity [Kg/m·s]	Molecular Weight [Kg/kmol]
Dry air	See Appendix A	1006.43	1.7894 × 10^−5^	28.966
Water vapor	0.0261	See Appendix A	1.34 × 10^−5^	18.01534

**Table 3:** The physical properties of the solids.

	k [W/m·K]	c [J/Kg·K]	Density [Kg/m^3^]
Tungsten	174	132	19,300
Silicon	40	700	2329
Silicon Dioxide	1.4	745	2200
Lead	35	0.13	11,342

**Figure A1:**
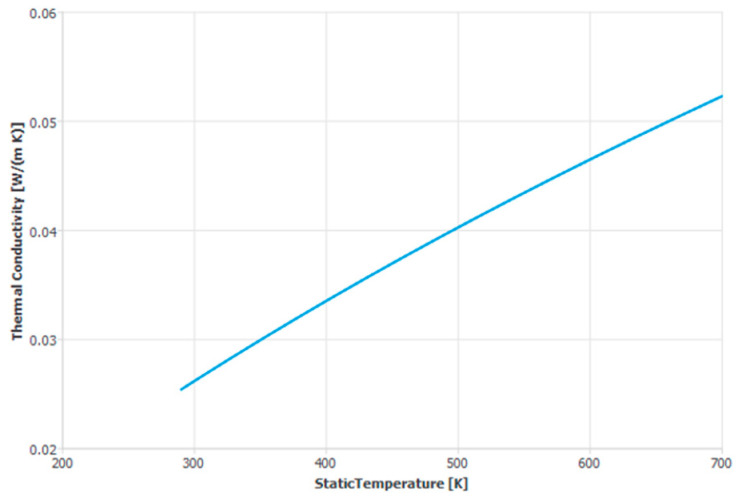
Air thermal conductivity as a function of the temperature.

**Figure A2:**
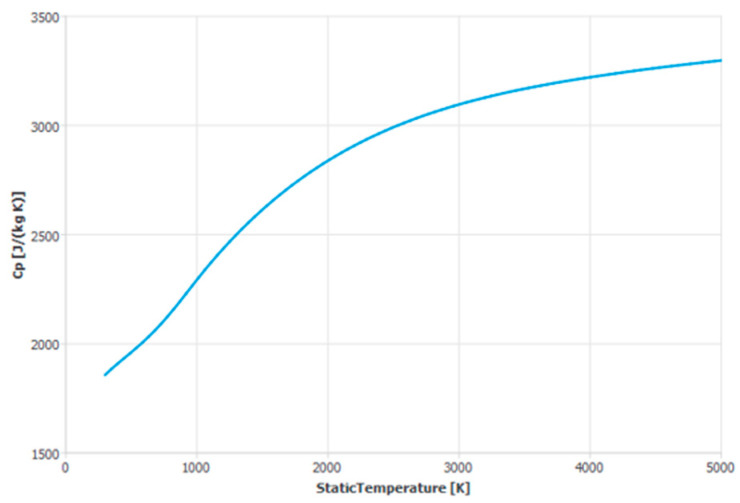
Specific heat capacity of water vapor as function of the temperature. Provided by the software [20] database.

## Data Availability

Data are contained within the article.

## Appendix A. Parameters for Simulation

The thermal conductivity of air as function of the temperature is not given by the software. It is taken from [16].

The specific heat capacity of the water vapor as function of the temperature:
